# Adherence to implementing physical distancing and other COVID-19 preventive measures and its barriers among adults in Arba Minch town, Southwest Ethiopia: A lesson learned from the pandemic

**DOI:** 10.1371/journal.pone.0315204

**Published:** 2024-12-31

**Authors:** Teklu Wegayehu, Tsegaye Yohannes, Chuchu Churko, Alemayehu Bekele, Mekuria Asnakew Asfaw

**Affiliations:** 1 Department of Biology, College of Natural and Computational Sciences, Arba Minch University, Arba Minch, Ethiopia; 2 Medical Laboratory Sciences, College of Medicine and Health Sciences, Arba Minch University, Arba Minch, Ethiopia; 3 Collaborative Research and Training Centre for Neglected Tropical Diseases, Arba Minch University, Arba Minch, Ethiopia; 4 MedStar Georgetown University Hospital, Washington, DC, United States of America; PLOS: Public Library of Science, UNITED STATES OF AMERICA

## Abstract

**Background:**

Globally, as of March 2024, the number of confirmed Coronavirus Disease 2019 (COVID-19) cases and deaths were over 774 million and seven million, respectively. Since there are no proven treatment in place against the disease, controlling strategy mainly rely on preventive measures. However, data on the extent of implementing physical distancing and other preventive measures during the pandemic of COVID-19 were inadequate in the study setting. This study is, therefore, documenting these gaps among people in Arba Minch town, South Ethiopia.

**Methods:**

We conducted a community-based cross-sectional survey and qualitative study at Arba Minch in June 2020. Quantitative data were collected using an interviewer-administered questionnaire and check-list from study participants (head of household or any adult ≥18 years old in a household) and analyzed using SPSS version 20. Qualitative data were collected using key informant interviews (KIIs) and analyzed by using a thematic approach.

**Results:**

Of the total surveyed adults (459), only 43.6% achieved above the mean score on adherence to implementing preventive measures of COVID-19. We found that 29.8% of participants kept physical distancing, only 37.7% of study participants had face-mask use practice, 20.5% had hand sanitizer use practice, 42.5% of participants avoided attendance in public gatherings, 44.7% stopped touching their nose, eye, and mouth, 55.6% practiced stay at home, and 60% had frequent hand washing practice. Besides, the main emerged barriers of adherence to implementing COVID-19 preventive measures were poverty, distrust of government, misbelief, lack of ownership, lack of attention and sustained actions, lack of ownership, socio-cultural influence, lack of coordination and leadership.

**Conclusions:**

The finding of this study suggests inadequate adherence to implementing COVID-19 preventive measures in adults in Arba Minch. Although inadequately implemented, a lot of lessons have been learned from COVID-19 pandemic preventive measures that would help in prevention and control of such a pantomimic disease happened.

## Introduction

The novel coronavirus disease 2019 (COVID-19) is a serious infectious disease, caused by the Severe Acute Respiratory Syndrome Coronavirus 2 (SARS-CoV-2) [1, 2]. On 30 January 2020, the World Health Organization (WHO) declared COVID-19 as a public health emergency of international concern on 30 January 2020 [3]. Human-to-human infection of SARS-CoV-2 occurs mainly through air droplets, close contact with infected persons, particularly mucus membranes secretions from nose, mouth, or eyes, contaminated surfaces. Some studies have also suggested digestive tract transmission [4, 5]. Elder people and those with underlying medical conditions, such as cardiovascular disease, diabetes, chronic respiratory disease, and cancer likely develop serious illnesses that may result in death [6–8].

Despite the level of advancement in the health system, COVID-19 were spread quickly across the United States, Europe, and South East-Asia early in the pandemic. As of 3 March 2024, over 774 million confirmed cases and over Seven million deaths have been reported globally [9]. Of these, Europe has reported the highest cases (36%) and death (32%); and Africa has reported the lowest case (1%) and death (2%). Although the number of confirmed novel COVID-19 cases reported in resource-poor settings is still relatively lower, this trend may be changed probably due to lack of adequate adherence to implementation of COVID-19 preventive measures. On the other hand, there is a high likelihood of the current number represents underestimates due to inadequate test accessibility [10].

After a decreasing trend since July 2021, case incidence rates in the African Region have begun to plateau, with over 20 000 new cases reported as of 7 November 2021. In Ethiopia, the first case was reported on March 13, 2020, at 48-year old Japanese in Addis Ababa [11]. Data for 5 February to 3 March 2024, Ethiopia has reported (40 new cases; <1 new case per 100 000) [9].

The WHO advises people to adhere to different preventive measures of the COVID-19 pandemic. According to the WHO recommendations, the best way to halt transmission of human-to-human is being well informed about the virus, how it spreads and adhering to the preventive measures adequately [12]. In response to the COVID-19 pandemic, the WHO along with its partners has been leading global coordination to hold the spread and reduce devastating impact of the COVID-19 pandemic [13].

Since the first incidence of the virus in Ethiopia, the country has been implementing unprecedented measures to control the rapid spread of the ongoing COVID-19 [14, 15]. Ethiopia initiated a screening program, established quarantine, and treatment centers in addition to community awareness and strong enforcement to control the spread of the virus. However, anecdotally, it has been observed that communities are neglecting physical distancing and other preventive measures of COVID-19. Moreover, there are few published studies that assess implementation of preventive measures of COVID-19 among the general population in Ethiopia [16–21]. This study is, therefore, aimed to investigate the extent of physical distancing and other preventive measures among people in Arba Minch town, Southwest Ethiopia to document the lesson learned from the pandemic.

## Methods

### Study setting

This study was conducted in Arba Minch, a town of Gamo Zone, which is located at 505kms south of Addis Ababa, the capital of Ethiopia. The town has 11 *kebeles* (the smallest unit of the government administration). Based on the 2007 census conducted by central statistical agency, the total projected population for 2020 is 120, 736 (60, 127 men and 60, 609 women) [22]. As any part of the country, community members in Arba Minch are at-risk for getting the coronavirus infection probably due to the existence of strong social interaction in the society which could favour the virus transmission rapidly. Since the first incidence of COVID-19 cases in Ethiopia, quarantine, and treatment centers were established in the town. Since the first incidence of confirmed COVID-19 cases in Ethiopia, awareness creations campaign has been done; and quarantine and treatment center have been established in the town.

### Study design and period

A mixed-study methods ─ combining a community-based cross-sectional survey and qualitative study was conducted at Arba Minch; from 15/06/2020–30/06/2020 to document the practice.

### Study population

The study population was the head of household or any adult ≥18 years old in the selected households who were residents and available during the survey period. Individuals were excluded from the study in the case when they were seriously ill and unable to provide information. For qualitative data, *kebele* administrators, managers, health office heads, head of health centers, hotel managers, and transport office heads were involved as key informants.

### Sample size and sampling technique

The sample size was determined using single proportion formula,

$n=\frac{{\left(Z_{\left(\frac{\alpha }{2}\right)}\right)}^{2}P(1-P)}{d^{2}}$, where, **p** is 50% (proportion of people implementing preventive measures), since there are no previous studies conducted in the study area; **Zα/2** is 1.96, the reliability coefficient of standard error at 5% level of significance, and desired degree of precision **(d)** of **5%**; the estimated sample size was **385**, and by adding **20%** non-response rate, the total computed sample size was 462. Study participants were selected using systematic random sampling technique from each *kebele* with consideration given to equal probability proportionate to sample size. For qualitative data, data were collected from 17 key informants of all *kebeles*. The number of key informants was determined based on information saturation.

### Study variables

Different variables were included in this study, such as socio-demographic and economic characteristics, source of information, knowledge, and perception on prevention and control of COVID-19, and hygiene-related factors. Implementation status of physical distance and other preventive measures were the main outcome variables.

### Data collection

Data were collected using house-to-house visits using interviewer administered-questionnaires and observation checklists. Data quality was ensured by developing, adapting, and pre-testing standardized tool; which is adapted from the WHO guidelines; training of data collectors and supervisors; and daily checking of consistency and accuracy of data. Data collectors and supervisors used face masks and alcohol-based hand rub and kept physical distancing to safeguard them and participants while they collect data collect. Qualitative data were collected using key informant interviews (KIIs).

### Data analysis

Data were edited, coded, and entered into Epidata version 4.4.2 and exported to SPSS version 25 software for analysis. Then, data were cleaned and frequencies and proportion analyses were done for the variables, and presented by figures and tables. For physical distancing, from each public gathering place, such as market, bank, church, *ekub*, hotels, bus station, and office distances between any two or more individuals were measured and study participants also reported their perceived practice of physical distancing. Differences in the implementation of preventive measures were assessed using the Chi-Square test (X^2^). Implementing preventive measures was measured using 12 questions and score was computed by counting values within a case. Qualitative data were analyzed using a thematic approach.

### Ethics statement

This study was reviewed and approved with the reference number of IRB/412/12 by the Institutional Research Ethics Review Board of College of Medicine and Health Sciences, Arba Minch University. Oral and written consents were received from sub-city administrators and heads of households before data collection started. Using information sheets and consent forms, written consent was obtained from study participants. The reason for taking written consent was to ensure participants are well informed about the study and agreed to involve in the study.

## Results

### Socio-demographic and economic characteristics

A total of 459 individuals participated in this study; which resulted in a response rate of 99.4%. Table 1 presents detail on socio-demographic and economic data. The mean number of individuals in a household was 4.9 (= ±1.95). Of the total participants, males were higher than females (56.4% versus 43.6%). Almost 32 (7%) respondents earned less than 1000 Ethiopian Birr (ETB) (~22.73 USD) per month, and 150 (32.7%) of them did not have hand washing facilities. Of the total participants, 86.5% accessed to COVID-19 related information from private television (TV), and 60.4% accessed from government TV. The remaining respondents got information from social media, friends, radio, family members, and town criers (Table 1).

**Table 1:** Socio-demographic and economic characteristics of study participants in Arba Minch town, June, 2020.

Characteristics	Category	Frequency	%
Sex	Female	200	43.6
	Male	259	56.4
Age (years)	18–29	86	18.7
	30–39	123	26.8
	40–49	126	27.5
	50–59	66	14.4
	≥ 60	58	12.6
Educational status	Cannot read and write	34	7.4
	Can read and write	24	5.2
	Grade 1–8	61	13.3
	Grade 9–12	103	22.4
	College and above	237	51.7
Occupation	Farmer	16	3.5
	Government employee	169	36.8
	Business (self)	136	29.6
	Unemployed	33	7.2
	Others*	105	22.9
Marital status	Single	42	9.2
	Married	376	81.9
	Divorced	22	4.8
	Windowed	19	4.1
Number of household members	<5	295	64.3
	≥5	164	35.7
Monthly income (USD)	<22.73	32	7.0
	22.73–68.16	112	24.4
	68.18–113.61	59	12.9
	113.64–136.34	102	22.2
	136.36–181.79	55	12.0
	181.82–227.25	35	7.6
	≥ 227.27	64	13.9
Housing condition	House or apartment with garden	18	3.9
	Condominium	10	2.2
	House or apartment in a building	10	2.2
	House in a fence where many people live	355	77.3
	Villa	44	9.6
	*Kebele* house	22	4.8
Obtain adequate water for hygiene	Yes	444	96.7
	No	15	3.3
Hand wash facility	Yes	309	67.3
	No	150	32.7
Soap available around hand wash facility (n = 309)	Yes	281	90.9
	No	28	9.1

^*^ = Housewife = 51; daily labourer = 54

### Socio-demographic characteristics of key informants

Participants in KIIs were adults (≥18 years) who play key role in the community and act as potential source of information regarding barriers to implementing COVID-19 preventive measures.

### Participant’s perception and other COVID-19 related information

Table 2 shows detail on perception and other COVID-19 related information among the study participants. Of the surveyed participants (459), nearly all (99.3%) were informed about COVID-19. However, only 128 (27.9%) responded that infected persons are the main source of infection, 339 (73.9%) knew COVID-19 symptoms, and 354 (77.1%) believed that COVID-19 can be prevented. In addition, 17 (3.7%) participants faced psychological violence while implementing preventive measures; 30 (6.5%) respondents had a history of in-country travel within the last 7 days of data collection, and 66 (14.4%) participants were worried about their health.

**Table 2:** Participant’s perception and other COVID-19 related information at Arba Minch, June, 2020 (n = 459).

Knowledge and perception related data	Category	Frequency	%
Have you been informed about COVID-19?	Yes	456	99.3
	No	3	0.7
Do you believe in effectiveness of preventive measure?	Yes	420	91.5
	No	39	8.5
How SARS-CoV-2 transmitted?	Air	143	31.2
	Water	3	0.6
	Infected person	128	27.9
	Contact	181	39.4
	I do not know	4	0.9
Knew COVID-19 symptoms	Yes	339	73.9
	No	120	26.1
Do washing hand with soap or applying alcohol based rub kill virus?	Yes	388	84.5
	No	67	14.6
	I do not know	4	0.9
How many seconds does hand washing recommended?	1 second	3	0.6
	5 seconds	28	6.1
	20–40 seconds	332	72.3
	60 seconds	20	4.4
	I do not know	76	16.6
Is SARS-CoV-2 can be prevented?	Yes	354	77.1
	No	94	20.5
	I do not know	11	2.4
Do you think vaccine available for COVID-19?	Yes	118	25.7
	No	303	66.0
	I do not know	38	8.3
Do you have underlying diseases?	Yes	89	19.4
	No	370	80.6
Are there family members with flu like symptom in the last 7 days?	Yes	39	8.5
	No	420	91.5
How worried your health these times?	Not at all	271	59.0
	Little worried	69	15.0
	Moderately	48	10.5
	Very much	66	14.4
	Extremely	5	1.1
Have you faced psychological violence due to practicing COVID-19 preventive measures?	No	442	96.3
	Yes	17	3.7
What means of transportation you use?	Public transport	283	61.6
	Own vehicle	53	11.6
	Rented	15	3.3
	On foot	108	23.5

### Status of implementing physical distancing

**Fig 1:**
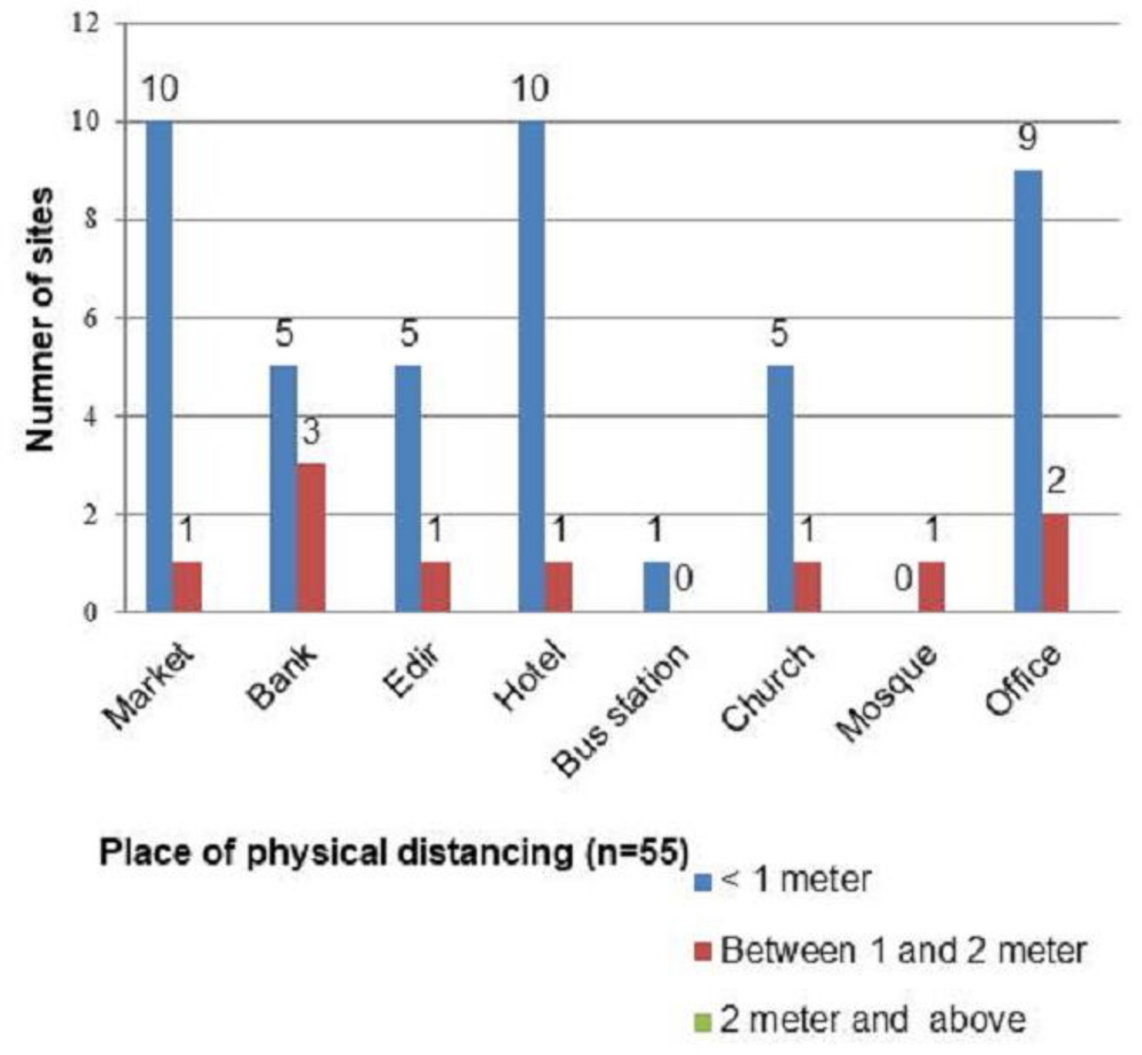
Physical distance between two or more individuals in different public gathering places of Arba Minch town, June, 2020 (n = 55).

Of the 55 surveyed public gathering places, the measured physical distances between any two or more people were less than 1 meter in 45 (81.8%) of places, and, surprisingly the recommended physical distance (at least 2 meters) was not kept in any of those places (Fig 1). On the other hand, of the total respondents (459), only 29.8% (137) of participants self-reported as they maintained at least a 2-meter distance outside their home (Table 3).

**Table 3:** Implementing other preventive measures by study participants in Arba Minch town, June, 2020.

Variables	Category		Frequency		%
Which preventive measure best to prevent COVID-19?	Face mask		46		10.0
	Wash hand		87		18.9
	Stay home		222		48.4
	Social distancing		98		21.4
	I do not know		6		1.3
Maintain at least 2 meter and above	Yes		137		**29.8**
	No		322		70.2
If at least 2 meter and above not maintained, do you wear facemask?	Yes		130		**40.4**
	No		192		59.6
Measure body temperature every two week	Yes		60		**13.1**
	No		399		86.9
Tested for COVID-19	Yes		11		2.4
	No		448		97.6
Stay at home					
Do you stay at home if going out side is not mandatory?	Yes		255		**55.6**
	Not		204		44.4
How difficult staying at home	Not at all		142		30.9
	Little		66		14.4
	Moderate		94		20.5
	Very difficult		116		**25.3**
	Extremely		41		8.9
**Hand washing**					
Hand wash practice to prevent COVID-19	Yes		423		**92.2**
	No		36		7.8
Frequency of hand in 24 hours (n = 423)	Rarely		4		0.9
	Sometimes		165		39.1
	Frequently		254		**60.0**
Wash hand after toilet	Yes		454		98.9
	No		5		1.1
Wash hand after touching any item	Yes		293		**63.8**
	No		166		36.2
Wash hand after touching your eye, nose or mouth	Yes		169		**36.8**
	No		290		63.2
Hand wash after work	Yes		449		**97.8**
	No		10		2.2
Wash hand before eating	Yes		458		99.8
	No		1		0.2
**Hand sanitizer**					
Practice of using hand sanitizer or alcohol based hand rub	Yes		326		**71.0**
	No		133		29.0
Frequency of using hand sanitizer (n = 326)	Rarely		20		6.2
	Sometimes		239		73.3
	Frequently		67		**20.5**
Disinfect phone when return to home	Yes		108		23.5
	No		351		76.5
Disinfect hand after you cough or sneeze	Yes		173		**37.7**
	No		286		62. 3
**Public gathering**					
Avoided going to public gathering place in the last 7 days		Yes		195	**42.5**
		No		264	57.5
**Face mask**					
If not avoided going to public gathering place, do you wear face mask? (N = 264)		Yes		166	**62.9**
		No		98	37.1
Wear face mask		Yes		173	**37.7**
		No		286	62.3
Type of face mask (N = 173)		Disposable		12	6.9
		Reusable cloth		140	80.9
		Professional mask		21	12.2
When do you wear face mask? (N = 173)		Some times when go out		52	30.1
		Every time when go out		115	66.5
		At work		6	3.4
If not use face mask, why? (N = 286)		No money		130	**45.5**
		I do not where to get		66	23.1
		Uncomfortable to use		84	29.4
		Not necessary		6	2.1
**Protect other people**					
Do you protect people around?		Yes		344	**74.9**
		No		115	25.1
Do you cover your mouth with elbow or cloth or mask when you cough or sneeze?		Yes		393	**85.6**
		No		66	14.4
Prefer home stay and isolate while having flue like symptoms		Yes		317	**69.1**
		No		142	30.9
Avoid hand shaking		Yes		410	**89.3**
		No		49	10.7
Number of people you met face-to-face within the last 24 hours		Zero		102	22.2
		20–40		31	6.8
		25–50		326	71.0
Seek medical treatment if gets flue like symptoms		Yes		368	**80.2**
		No		91	19.8
Stopped touching eye, nose, and mouth		Yes		205	**44.7**
		No		254	55.3
Perceived self-evaluation on preventive measure		≤ 6		168	**36.6**
		7 and above		291	63.4
Mean score on preventive measures		≤ 6		259	56.4
		7 and above		200	**43.6**
		No		254	55.3

**Fig 2:**
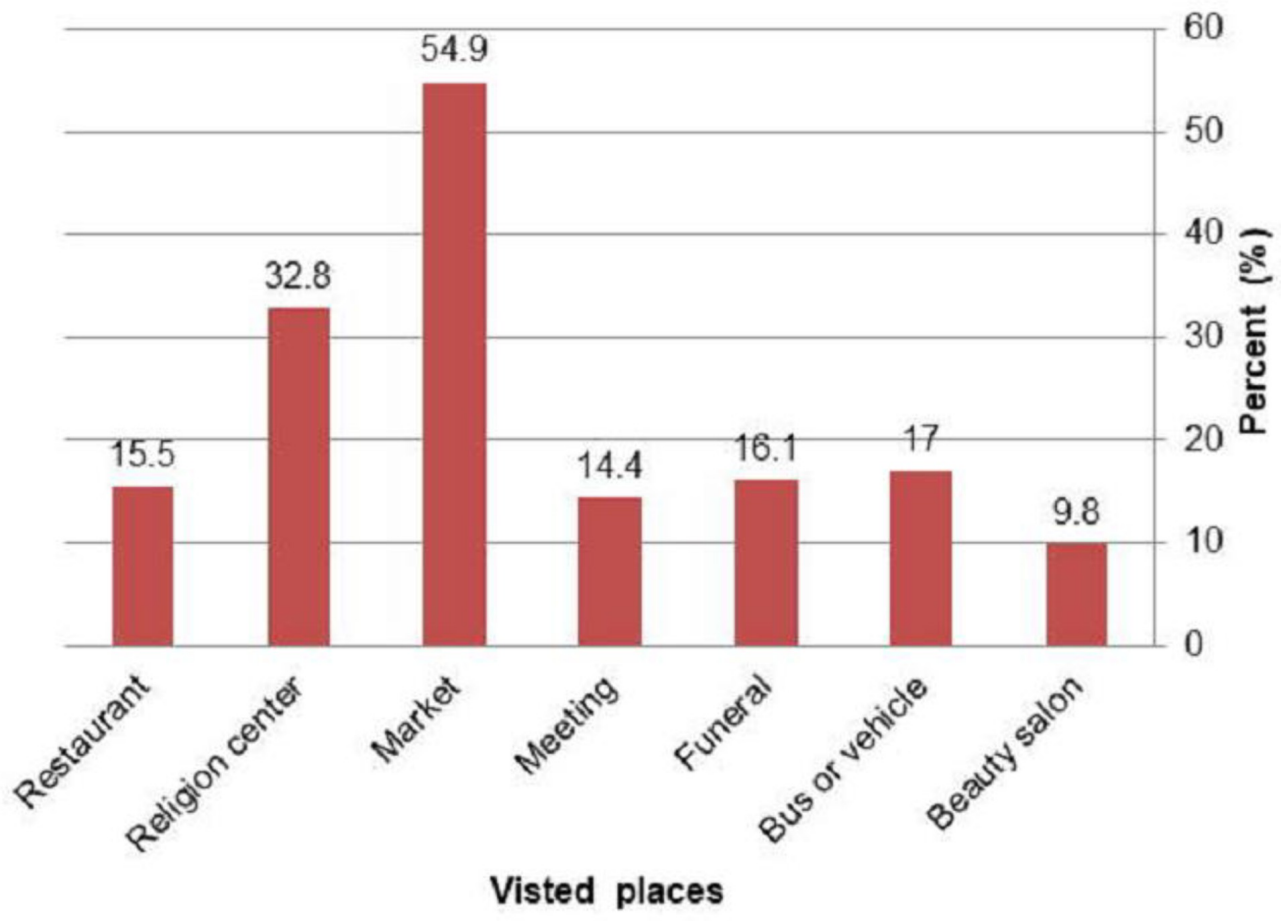
Public gathering places visited by participants in the last seven days before data collection in Arba Minch town, June, 2020.

Concerning to visiting crowded places, 252 (54.9%) of participants visited market and 32.8% went to religious centers (Churches and Mosques) in the last seven days ahead of data collection (Fig 2).

### Status of implementing other preventive measures

We used 12 questions to assess the implementation of preventive measures against COVID-19. In total, 200 (43.6%) participants achieved above the mean score (6±1.97) on preventive measures. Of the surveyed individuals, only 173(37.7%) had face mask use practice, 67

(20.5%) had frequent hand sanitizer use practice, and 60 (13.1%) were measuring their body temperature every two weeks. Moreover, 195 (42.5%) avoided going to the public gathering places in the last 7 days; 205 (44.7%) stopped touching their nose, eye and mouth; and 255 255(55.6%) practiced stay-at-home if going outside is not mandatory, and 254 (60%) had frequent hand washing practice. In addition, the majority, 306 (66.7%) practiced covering their mouth and nose while coughing or sneezing with cloth or tissue; 317 (69.1%)practiced isolating themselves while having flue like symptoms; and mouth, 313 (68.2%) had treatment-seeking behavior if they experience flu like symptoms; and 410 (89.3%) avoided hand shaking. Among those who did not use a face mask, the main mentioned reason was not having money, 130 (45.5%) to purchase the mask (Table 3).

### Difference in implementing preventive measures

A range of numerical differences in implementing COVID-19 preventive measures by socio-demographic variables was noted among study participants. Slightly more females (73%) had a practice of keeping physical distance compared to males (68%). Similarly, slightly more females (63%) had a practice of keeping physical distance compared to males (61.8%). In addition, individuals aged 40–49 had higher practice of keeping physical distancing and face mask use practice. Moreover, females had a higher practice of hand washing and stay at home practice. On the contrary, the difference in availability of hand washing was statistically significant with hand washing practice at p-value <0.05 (Table 4).

**Table 4:** Socio-demographic characteristics related difference in implementing selected preventive measures by study participants toward COVID-19 (n = 459).

Variables	Category	Preventive measures					
		Practice physical distancing			Use face mask		
		Yes	No	X^2^ (P-value)	Yes	No	X^2^ (P-value)
		N (%)	N (%)		N (%)	N (%)	
**Sex**	Male	176 (68.0)	83 (32.0)	**1.373 (0.241)**	160 (61.8)	99 (38.2)	**0.072 (0.788)**
	Female	146(73.0)	54 (27.0)		126 (63.0)	74 (37.0)	
**Age**	18–29	59 (68.6)	27 (31.4)	**4.167 (0.384)**	59 (68.6)	27 (31.4)	**2.817 (0.589)**
	30–39	86 (69.9)	37 (30.1)		72 (58.5)	51 (41.5)	
	40–49	94 (74.6)	32 (25.4)		76 (60.3)	50 (39.7)	
	50–59	48 (72.7)	18 (27.3)		39 (59.1)	27 (40.9)	
	≥ 60	35 (60.3)	23 (39.7)		40 (69.0)	18 (31.0)	
**Educational status**	Cannot read and write	27 (79.4)	7 (20.6)	**2.836 (0.586)**	26 (76.5)	8 (23.5)	**8.928 (0.06)**
	Can read and write	15 (62.5)	9 (37.5)		17 (70.8)	7 (29.2)	
	Grade 1–8	45 (73.8)	16 (26.2)		38 (62.3)	23 (37.7)	
	Grade 9–12	73 (70.9)	30 (29.1)		71 (68.9)	32 (31.1)	
	College and above	162 (68.4)	75 (31.6)		134 (56.5)	103 (43.5)	
**Occupation**	Farmer	13 (81.2)	3 (18.8)	**5.243 (0.263)**	13 (81.3)	3 (18.8)	**2.817 (0.58)**
	Government employee	113 (66.9)	56 (33.1)		102 (60.4)	67 (39.6)	
	Business (self)	95 (69.9)	41 (30.1)		86 (63.2)	50 (36.8)	
	Unemployed	28 (84.8)	5 (15.2)		20 (60.6)	13 (39.4)	
	Others*	73 (69.5)	32 (30.5)		65 (61.9)	40 (38.1)	
**Variable**	**Category**	**Hand wash regularly**			**Stay-at-home**		
		**Yes**	**No**	**X**^**2**^ **(P-value)**	**Yes**	**No**	**X**^**2**^ **(P-value)**
		**N (%)**	**N (%)**		**N (%)**	**N (%)**	
**Sex**	Male	19 (7.3)	240 (92.3)	**0.212 (0.646)**	160 (61.8)	99 (38.2)	0.072 (0.788)
	Female	17 (8.5)	183 (91.5)		126 (63.0)	74 (37.0)	
**Age**	18–29	10 (11.6)	76 (88.4)	**5.024 (0.285)**	59 (68.6)	27 (31.4)	**3.795 (0.434)**
	30–39	12 (9.8)	111 (90.2)		72 (58.5)	51 (41.5)	
	40–49	6 (4.8)	120 (95.2)		76 (60.3)	50 (39.7)	
	50–59	3 (4.5)	63 (95.5)		39 (59.1)	27 (40.9)	
	≥ 60	5 (8.6)	53 (91.4)		40 (69.0)	18 (31.0)	
**Educational status**	Cannot read and write	2 (5.9)	32 (94.1)	**6.850 (0.144)**	26 (76.5)	8 (23.5)	**8.928 (0.063)**
	Can read and write	4 (16.7)	20 (83.2)		17 (70.8)	7 (29.2)	
	Grade 1–8	1 (1.6)	60 (98.4)		38 (62.3)	23 (37.7)	
	Grade 9–12	7 (6.8)	96 (93.2)		71 (68.9)	32 (31.1)	
	College and above	22 (9.3)	215 (90.7)		134 (56.5)	103 (43.5)	
**Occupation**	Farmer	1 (6.3)	15 (93.8)	**7.061 (0.133)**	13 (81.3)	3 (18.8)	**2.817 (0.589)**
	Government employee	19 (11.2)	150 (88.8)		102 (60.4)	67 (39.6)	
	Business (self)	5 (3.7)	131 (96.3)		86 (63.2)	50 (36.8)	
	Unemployed	4 (12.1)	29 (87.9)		20 (60.6)	13 (39.4)	
	Others*	7 (6.7)	98 (93.3)		65 (61.9)	40 (38.1)	
**Hand washing facility**	Yes	18 (5.8)	291 (94.2)	**5.327 (0.021)** ^*^	-	-	**-**
	No	18 (12)	132 (88)		-	-	

^*^ **= Housewife = 51 and daily labourer = 54**

^*^ **=** P-value significant at <0.05

### Barriers for adherence to implementing COVID-19 preventive measures

The main identified barriers for adherence to implementing COVID-19 preventive measures among adults were poverty, lack of attention and sustained actions, distrust of government, misbelief, lack of ownership, lack of ownership, socio-cultural factors, lack of coordination, and leadership.

#### “Poverty”

“…Individuals in our community are very poor primarily due to high unemployment rate; as a result, people say ‘better to die from the coronavirus than from hunger’…” [Village chief]

#### “Lack of attention and sustained action”

“…Implementing prevention activity among community members was good when people heard occurrence of the virus for the first time, but these days people thought as coronavirus has been eliminated ─ community members have neglected implementing the preventive measures. We failed to adhere with the COVID-19 preventive measures…” [Village chief…]

“…Physical distancing is difficult to implement in our community, especially in market and funeral places…” [*Ekub* leader]

“…Even public servant organization do not give the due attention to implement COVID-19 preventive measures…” [Health facility head]

“…Now I lose hope to prevent coronavirus because everybody has stopped to adhere with COVID-19 preventive measures…” [Village chief]

**“Distrust of government”** ─ “…There is no coronavirus ─ there is politics…” [Community member]

“Misbelief”.

“…My creature can save me; I do not need to implement the preventive measures…” [Community member]

“…Many individuals believe that COVID-19 came to us from God because of our sin…”

[Village chief]

“Lack of ownership and community engagement”.

“…People wear a face mask only when they come to the office; surprisingly even there are few individuals that share face masks by taking from colleagues to enter into office. This mainly due to lack of ownership, awareness and community engagement…” [Village chief]

“Socio-cultural influence”.

“…People in our community have a culture of eating together ─ sharing on a single dish. So, our cultures also adversely affect adherence to COVID-19 preventive measures…” [Hotel manager]

“…There is strong social interaction in our community, and it is difficult to keep physical distance…” [Village chief]

“Lack of leadership and coordination”.

“…Even leaders are not implementing the preventive measures; they have to act as a model and should coordinate activities…” [Village chief]

## Discussion

Evidence of this study provides insights on adherence to the recommended COVID-19 preventive measures among adults in Arba Minch, Southwest Ethiopia. The study showed that adherence to implementing COVID-19 preventive measures was found to be low in adults. Besides, poverty, lack of attention and sustained action, distrust of government, misbelief, lack of ownership, lack of sustained action, lack of ownership, socio-cultural factors, lack of coordination and leadership were identified as the main barriers for adherence to preventing measures of coronavirus. While almost all participants (99.3%) were informed on COVID-19, we found that only 43.6% of participants achieved above the mean score (6±1.97) regarding preventive measures. The low adherence of participants to the COVID-19 preventive measures might be due to a lack of community engagement, ownership, and participation in playing a part to tackle the COVID-19 pandemic.

In the current study, only 29.8% of participants self-reported as they kept recommended physical distance (at least 2 meters) outside their home. Besides, the possible reason for the low implementation of physical distancing in this study is the strong socio-cultural interaction that exists in society. Consistent with this finding, a facility based study conducted in another part of Ethiopia (Jimma) showed a slightly higher practice of avoiding physical proximity [19]. Our finding is much lower than a finding among Vietnamese people where 88.2% of the participants adhere to the physical distancing rule [23]. Likewise, Block and colleagues ascribed that 67% of African Americans adhere to maintain physical distancing [24]. The possible reason for low implementation of physical distancing in our study is probably due to the strong social interaction norms that exist in the society.

In this study, only 37.7% of participants had face mask use practice when leaving out home, which is relatively higher than the face mask use practice among Residents of Dirashe District (20.5%). Southern Ethiopia [16]. Participants in this study had low face mask use practice when leaving out home in comparison with face mask use practice of study participants in a study done in Vietnamese people (99.5%), Malaysia (51.2%), Saudi Arabia (75%) and China (98%) [23, 25–27]. The lower practice of face masks in our study might be due to the low economic status of study participants to purchase face masks, as justified by data of our study.

Finding of the current study showed that only 20.5% and 60% of participant had frequent use of hand sanitizer and hand washing practice, respectively. Likewise, Bedane and colleagues ascribed only 11.1% of Jimma town inhabitants frequently use hand sanitizer [18]. The reason behind for the low utilization of hand sanitizer in our study might be related to lack of access to hand sanitizer at an affordable cost. On the other hand, findings of studies conducted in Vietnamese people (97.4%), African Americans (72%), Malaysia (87.8%) and Philippines (82.2%) revealed much better hand washing practice [23–25, 28]. The inadequate hand washing practice observed in this study could be due to lack of sustainable social behavioral change communication (SBCC).

In this study, less than half (42.5%) of participants avoided going to public gathering places in the last 7 days, and many people move to market areas to purchase their groceries. Inconsistent with this finding, a study conducted in Jimma town (Ethiopia) [18] and Malaysia [25] demonstrated a higher avoidance of going to public place. This result might be due to the fact that strong social interaction norms exist in society, and this finding is also justified by our data.

With regard to stopping touching nose, eye and mouth practice, in our study, 44.7% of participants stopped touching their nose, eye and mouth. The finding is also lower than that of the African Americans who avoid touching face by 55% [24]. This finding indicated that still more intervention is required to bring behavioral change. This study demonstrated that more than half of participants practiced stay at home preventive measure. However, data of our study showed that a substantial number of participants mentioned that stay at home is very challenging as a result of economic problems, which force people to go outside their home to look for their daily breads.

Data of our study revealed that 66.7% of respondents had practice of covering their mouth and nose while coughing or sneezing. This is much lower than practice of cover mouth and nose during coughing or sneezing by Vietnamese people (94.9%) [23]. Inadequate mouth and nose covering while coughing or sneezing with cloth, mask or tissue observed in this study could be due to lack of SBCC. In the current study, a substantial of participants had treatment-seeking behavior if they experience flue like symptoms. This might be due to people having high fear of the virus as it could result in death. Moreover, 69.1% of participants practiced isolating themselves while having flue like symptoms. Consistent with this finding, a bi-national study conducted in Africa (Nigeria and Egypt) revealed that “as many as 96% of study participants practiced self-isolation and social distancing” [29].

In this study, although high proportion of participants avoided handshaking practice, the percentages of people who believe infected people are the main source of infection was low. This might be lack of awareness related to coronavirus transmission and prevention. On the contrary, a lower practice of handshaking was observed in a study conducted in another part of Ethiopia (53.8%) [19].

Furthermore, poverty, lack of attention, distrust of government, misbelief, lack of ownership, lack of attention and sustained actions, lack of ownership, socio-cultural influence, lack of coordination and leadership were identified as main barriers of adherence to implementing COVID-19 preventing measures. These challenges could be addressed by integrating health information related to COVID-19 in the exiting health extension program of community health platform of Ethiopia. Moreover, to the best of our knowledge scarce evidence exists in this regard, and we could not discuss the implication of these study findings in comparison with other studies’ findings.

Indeed, the pandemic of COVID-19 was a public health as well as socio-economic problem around the world. We lost our relatives, colleagues and friends by death. Its burden has been reflected on communicable and non-communicable diseases as the health facilities were focused on COVID-19 prevention. The global economy was seriously impacted to the extent of shut the system. However, with these all-observable challenges and problems our study found out that only 43.6% of participants achieved above the mean score (6±1.97) on preventive measures. The current study, in fact, exposes the need for more comprehensive education programs with focus on consistency of information for newly emerging pathogens like COVID-19.

The main strength is that we possibly assessing community’s adherence to the recommended preventive measures of COVID-19 pandemic at community level and its barriers which address an important national research priority. While interpreting data presented in this study, the following limitations need to be considered. First, findings are relying on self-reported practices of participants. Second, participant may report for the purpose of social desirability.

## Conclusions

The finding of this study suggests that inadequate adherence to implementing COVID-19 preventive measures in adults in Arba Minch, Southwest Ethiopia. Thus, lesson learned from the preventive measures of COVID-19 would help to take action, if such a pandemic will be happened.
