# Supplementary material for: Adherence to implementing physical distancing and other COVID-19 preventive measures and its barriers among adults in Arba Minch town, Southwest Ethiopia: A lesson learned from the pandemic
Source: PLoS One. 2024 Dec 31;19(12):e0315204. doi: 10.1371/journal.pone.0315204 (PMC11687725; doi:10.1371/journal.pone.0315204)
Supplement: Attachment — Submitted filename: Response to Reviewers.docx [file pone.0315204.s001.docx]

**Response to Reviewers**

First of all, we would like to extend our thankfulness to Editor’s and reviewers for their detailed appraisal. All the comments and suggestions given are still helpful and constructive. We accepted all the corrections, answered the questions, and tried to elaborate some sections. The amendments due to reviewers’ comments and suggestions; and major revisions we have made are highlighted by red color. Language editions are also made at different sections. Point-by-point response and elaborations to the two reviewers are given below.

Thank you!

**-------------------------//--------------------------**

**To Editor**

‘It has come to our attention that the manuscript seems to be a resubmission of the manuscript "[PONE-D-20-24749”, which was previously considered by the journal. It is a requirement by the journal that any manuscript resubmitted after a previous rejection is accompanied by a rebuttal letter outlining the response to the original reviewers' comments, so that the editors can establish whether the original concerns have been addressed. Can you therefore please provide us with a point-by-point response to the comments provided by the original reviewers, outlining the revisions carried out since the original submission’.

**Response:** Thank you so much for your clarification; and apology for the delay. The mistake we made is due to disconnection between the former 1^st^ author and co-authors. Later we discussed and managed the problem (see the changes made in the title page).

**General comments**

We have made revision to literature to place the study within the larger context of the scholarly literature. This has been indicated in the background of the abstract, introduction [paragraph 2,3 and 5] and discussion section. As a result, the intext citation and the list of reference is also updated. We have also made language edition in different section of the MS which is indicated in track change.

**Reviewer #2:**

The authors have done a wonderful job at improving the manuscript, particularly in the areas of clarity of writing and delivery of key messages. Just a few remaining comments from me.

1. Minor spelling error in line 142 of clean version of the manuscript – should be “tables” instead of “tales”?

2. Please provide a short half-sentence or so to clarify why it is “surprising” that study participants in China demonstrated “as high as 98% of respondents had face mask use practice” (Discussion, line 320)

3. The addition of qualitative data is thoughtful. It is important and valuable to have identified barriers to implementing COVID-19 preventive measures, but there is limited discussion about the nature of the barriers and implications for how any stakeholder may begin addressing them. For example, the barriers identified seem to point to deeply ingrained cultural behaviors and other issues at the institutional level, and will require action beyond the provision of health information (as suggested in lines 356-367). While I understand it may be outside of the scope of this study to address this fully, the authors should acknowledge the complexities of improving adherence to preventive measures and a need for a fuller investigation of the barriers raised in this study.

**Response:** We appreciated the reviewer’s comments, and accordingly amendment has been made in the revised manuscript.

The typographic error is corrected [see line 168]; the exaggeration word “surprising’ has been deleted [see line 357, in the discussion section] because use of the term has no relevance. The main barriers of adherence to implementing COVID-19 preventive measures has been explained [see line 412 in the discussion; or paragraph 13]

**Reviewer #3:**

All above questions from reviewers were answered. Correction regarding manuscript has been done. This study will definitely shows need of adherence to the covid appropriate behaviors.

**Response:** Thank you so much for accepting our response in the former replay!

**Reviewer #4:**

Authors have done study on Adherence to implementing physical distancing and other COVID-19 for preventive measures and its barriers among adults in Arba Minch town, Southern Ethiopia. Original article based mixed-study methods ─ combining a community-based cross-sectional survey and qualitative study at Arba Minch town is interesting to read.

Some comments:

1.Many of findings are similar whether data analysis was sub analysis

1 in community 2 nd on in hospitalized patients

Similar article on similar methods are available Bante A, Mersha A, Tesfaye A, Tsegaye B, Shibiru S, Ayele G, Girma M. Adherence with COVID-19 Preventive Measures and Associated Factors Among Residents of Dirashe District, Southern Ethiopia. Patient Prefer Adherence. 2021;15:237-249

https://doi.org/10.2147/PPA.S293647

2Kebede Y, Yitayih Y, Birhanu Z, Mekonen S, Ambelu A (2020) Knowledge, perceptions and preventive practices towards COVID-19 early in the outbreak among Jimma university medical center visitors, Southwest Ethiopia. PLoS ONE 15(5): e0233744. https://doi.org/10.1371

2. Data was collected in 2020. Vaccines were not available at that time against covid 19

3 References : citation not proper

1 Gorbalenya AE, Baker SC, Baric R, Groot RJ, Drosten C, Gulyaeva AA, Haagmans 373 BL, Lauber C, Leontovich AM, Neuman BW, Penzar D. Severe acute respiratory 374 syndrome-related coronavirus: The species and its viruses–a statement of the Coronavirus Study Group

2. Hotez PJ, Bottazzi ME, Singh SK, Brindley PJ, Kamhawi S. Will COVID-19 become 402 the next neglected tropical disease?

Article needs revision & correction & referencing, formatting

**Response:** The authors appreciated the reviewer’s comments regarding referencing and formatting among others. Accordingly, amendment has been made in different sections of the revised manuscript.

As I mentioned earlier, literature review has been made to place the study within the larger context of the scholarly literature. This has been indicated in the background of the abstract, introduction [paragraph 2,3 and 5] and discussion section. As a result, the intext citation and the list of reference is also updated. Hence, the studies conducted in Ethiopia [Derashe, Jimma, Jigjaga, SNNPR] and abroad [China, Vietna, Nigeria Egypt, Saudi etc..] have been included in the revision.

We have also made language edition in different section of the MS which is indicated in track change. The numbering of tables from Table 3 -5 has been corrected.

--------------------//--------------------
